# Supplementary material for: DPP8/DPP9 inhibition elicits canonical Nlrp1b inflammasome hallmarks in murine macrophages
Source: Life Sci Alliance. 2019 Feb 4;2(1):e201900313. doi: 10.26508/lsa.201900313 (PMC6362307; doi:10.26508/lsa.201900313)
Supplement: Supplementary file 1 [file LSA-2019-00313_TableS1.docx]

**Supplementary Table 1. Parameters used for calculation and the resulting concentrations of residual inhibitory activity of VBP and cVBP in cell lysates or medium.** BMDMs were mock-treated or received 10 µM of each inhibitor for 15min. Cell lysates and supernatants were quantified for their level of DPP9 inhibition, as described in Methods. The parameters included are: *y*: value of the residual activity compared to a non-inhibited sample; IC_50_^:^ half maximum inhibitory concentration.

| **Inhibitor** | **Parameter** | **Value** |
| --- | --- | --- |
| VBP | Medium | |
|  | Y-range | 1.03 ± 0.01 |
|  | Ic_50_ (nM) | 15.3 ± 0.8 |
|  | Slope factor | 1.39 ± 0.09 |
|  | Background | -0.01 ± 0.01 |
|  | Inhibitor in medium (µM) | 1.5 |
|  | Inhibition (%) | 100 |
|  | Cell lysate | |
|  | Y-range | 1.05 ± 0.02 |
|  | Ic_50_ (nM) | 10.0 ± 0.6 |
|  | Slope factor | 1.30 ± 0.09 |
|  | Background | -0.01 ± 0.01 |
|  | Inhibitor in lysate (µM) | 0.06 |
|  | Inhibition (%) | 93 |
| cVBP | Medium | |
|  | Y-range | 1.02 ± 0.02 |
|  | Ic_50_ (µM) | 1.70 ± 0.08 |
|  | Slope factor | 1.33 ± 0.08 |
|  | Background | -0.01 ± 0.01 |
|  | Inhibitor in medium (µM) | 3.2 |
|  | Inhibition (%) | 71 |
|  | Cell lysate | |
|  | Y-range | 1.04 ± 0.03 |
|  | Ic_50_ (µM) | 1.6 ± 0.1 |
|  | Slope factor | 1.4 ± 0.2 |
|  | Background | -0.02 ± 0.02 |
|  | Inhibitor in lysate (µM) | 0.3 |
|  | Inhibition (%) | 2.7 |
