# Supplementary material for: DPP8/DPP9 inhibition elicits canonical Nlrp1b inflammasome hallmarks in murine macrophages
Source: Life Sci Alliance. 2019 Feb 4;2(1):e201900313. doi: 10.26508/lsa.201900313 (PMC6362307; doi:10.26508/lsa.201900313)
Supplement: Supplementary file 2 [file LSA-2019-00313_TableS2.docx]

**Supplementary Table 2. Parameters used for calculation and the resulting concentrations of residual inhibitory activity of sitagliptin, UAMC39, UAMC1110 and KYP-2047** **in cell lysates or medium.** BMDMs were mock-treated or received 10 µM of each inhibitor for 15min. Cell lysates and supernatants were quantified for their level of inhibition of their target enzyme, as described in Methods. The parameters included are: *y*: value of the residual activity compared to a non-inhibited sample; IC_50_^:^ half maximum inhibitory concentration.

| **Inhibitor** | **Parameter** | **Value** |
| --- | --- | --- |
| sitagliptin | Medium | |
|  | Y-range | 1.02 ± 0.01 |
|  | Ic_50_ (nM) | 61 ± 3 |
|  | Slope factor | 1.03 ± 0.04 |
|  | Background | 0.005 ± 0.007 |
|  | Inhibitor in medium (µM) | 11.7 |
|  | Inhibition (%) | 99 |
|  | Cell lysate | |
|  | Y-range | 1.00 ± 0.01 |
|  | Ic_50_ (nM) | 40 ± 2 |
|  | Slope factor | 1.02 ± 0.04 |
|  | Background | 0.004 ± 0.007 |
|  | Inhibitor in lysate (µM) | 0.8 |
|  | Inhibition (%) | 95 |
| UAMC39 | Medium | |
|  | Y-range | 98 ± 2 |
|  | Ic_50_ (nM) | 9.8 ± 0.6 |
|  | Slope factor | 1.4 ± 0.1 |
|  | Background | -1 ± 1 |
|  | Inhibitor in medium (µM) | 52/45 |
|  | Inhibition (%) | 100 |
|  | Cell lysate | |
|  | Y-range | 90 ± 5 |
|  | Ic_50_ (nM) | 6 ± 1 |
|  | Slope factor | 1.3 ± 0.3 |
|  | Background | -1 ± 4 |
|  | Inhibitor in lysate (µM) | 120/66 |
|  | Inhibition (%) | 100 |
| UAMC1110 | Medium | |
|  | Y-range | 1.35 ± 0.03 |
|  | Ic_50_ (nM) | 0.52 ± 0.04 |
|  | Slope factor | 1.2 ± 0.1 |
|  | Background | -0.01 ± 0.02 |
|  | Inhibitor in medium (µM) | 8.8 |
|  | Inhibition (%) | 100 |
|  | Cell lysate | |
|  | Y-range | 1.33 ± 0.02 |
|  | Ic_50_ (nM) | 0.46 ± 0.02 |
|  | Slope factor | 1.21 ± 0.07 |
|  | Background | -0.02 ± 0.01 |
|  | Inhibitor in lysate (µM) | 0.4 |
|  | Inhibition (%) | 100 |
| KYP-2047 | Medium | |
|  | Y-range | 0.94 ± 0.02 |
|  | Ic_50_ (nM) | 3.0 ± 0.3 |
|  | Slope factor | 0.96 ± 0.07 |
|  | Background | 0.003 ± 0.011 |
|  | Inhibitor in medium (µM) | 8.4 |
|  | Inhibition (%) | 100 |
|  | Cell lysate | |
|  | Y-range | 1.08 ± 0.01 |
|  | Ic_50_ (nM) | 8.6 ± 0.4 |
|  | Slope factor | 1.10 ± 0.05 |
|  | Background | -0.003 ± 0.008 |
|  | Inhibitor in lysate (µM) | 1.1 |
|  | Inhibition (%) | 100 |
